# The Incoherent Fluctuation of Folate Pools and Differential Regulation of Folate Enzymes Prioritize Nucleotide Supply in the Zebrafish Model Displaying Folate Deficiency-Induced Microphthalmia and Visual Defects

**DOI:** 10.3389/fcell.2021.702969

**Published:** 2021-06-29

**Authors:** Tsun-Hsien Hsiao, Gang-Hui Lee, Yi-Sheng Chang, Bing-Hung Chen, Tzu-Fun Fu

**Affiliations:** ^1^The Institute of Basic Medical Science, College of Medicine, National Cheng Kung University, Tainan, Taiwan; ^2^Department of Ophthalmology, College of Medicine, National Cheng Kung University Hospital, Tainan, Taiwan; ^3^Department of Ophthalmology, College of Medicine, National Cheng Kung University, Tainan, Taiwan; ^4^Department of Biotechnology, Kaohsiung Medical University, Kaohsiung, Taiwan; ^5^Department of Medical Research, Kaohsiung Medical University Hospital, Kaohsiung, Taiwan; ^6^Center for Biomarkers and Biotech Drugs, Kaohsiung Medical University, Kaohsiung, Taiwan; ^7^Department of Biological Sciences, National Sun Yat-sen University, Kaohsiung, Taiwan; ^8^Department of Medical Laboratory Science and Biotechnology, College of Medicine, National Cheng Kung University, Tainan, Taiwan

**Keywords:** folate, one-carbon metabolism, eye diseases, zebrafish, microphthalmia, *mthfd1L*

## Abstract

**Objective:**

Congenital eye diseases are multi-factorial and usually cannot be cured. Therefore, proper preventive strategy and understanding the pathomechanism underlying these diseases become important. Deficiency in folate, a water-soluble vitamin B, has been associated with microphthalmia, a congenital eye disease characterized by abnormally small and malformed eyes. However, the causal-link and the underlying mechanism between folate and microphthalmia remain incompletely understood.

**Methods:**

We examined the eye size, optomotor response, intracellular folate distribution, and the expression of folate-requiring enzymes in zebrafish larvae displaying folate deficiency (FD) and ocular defects.

**Results:**

FD caused microphthalmia and impeded visual ability in zebrafish larvae, which were rescued by folate and dNTP supplementation. Cell cycle analysis revealed cell accumulation at S-phase and sub-G1 phase. Decreased cell proliferation and increased apoptosis were found in FD larvae during embryogenesis in a developmental timing-specific manner. Lowered methylenetetrahydrofolate reductase (*mthfr*) expression and up-regulated methylenetetrahydrofolate dehydrogenase (NADP^+^-dependent)-1-like (*mthfd1L*) expression were found in FD larvae. Knocking-down *mthfd1L* expression worsened FD-induced ocular anomalies; whereas increasing *mthfd1L* expression provided a protective effect. 5-CH_3_-THF is the most sensitive folate pool, whose levels were the most significantly reduced in response to FD; whereas 10-CHO-THF levels were less affected. 5-CHO-THF is the most effective folate adduct for rescuing FD-induced microphthalmia and defective visual ability.

**Conclusion:**

FD impeded nucleotides formation, impaired cell proliferation and differentiation, caused apoptosis and interfered active vitamin A production, contributing to ocular defects. The developmental timing-specific and incoherent fluctuation among folate adducts and increased expression of *mthfd1L* in response to FD reflect the context-dependent regulation of folate-mediated one-carbon metabolism, endowing the larvae to prioritize the essential biochemical pathways for supporting the continuous growth in response to folate depletion.

## Graphical Abstract

Folate deficiency lowered intracellular 10-CHO-THF and 5-CH3-THF levels, interfered nucleotide formation, impaired cell proliferation, induced apoptosis and impeded vitamin A supply, leading to microphthalmia and obstructed visual ability.

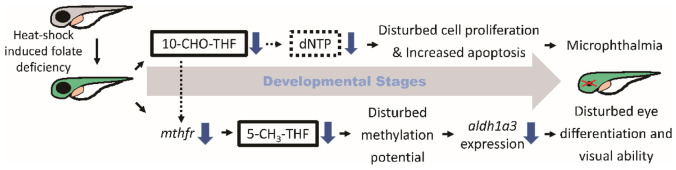

## Introduction

Microphthalmia is a congenital ocular disorder with the phenotypic characteristics of abnormally small and anatomically malformed eyes. The prevalence of congenital microphthalmia was approximately 1–2 per 10,000 new births (Stoll et al., 1992; Parker et al., 2010; Dharmasena et al., 2017). Although seldom fatal, most congenital ocular defects, including microphthalmia and impaired visual ability, cause life-long suffering to the affected individuals and a tremendous burden to families. Currently, the etiology for most congenital ocular diseases remains incompletely understood but is considered multifactorial. Among the reported risk factors, folate deficiency (FD) has been suggested as a potential environmental cause of microphthalmia. Studies showed that rodents with FD or disturbed folate-mediated one-carbon metabolism (FOCM) displayed anophthalmia and microphthalmia (Armstrong and Monie, 1966; Shaw et al., 2007; Maestro-de-las-Casas et al., 2013; Leung et al., 2017). However, how FD leads to these eye diseases and whether folate supplementation provides effective protection remain elusive.

Folate, also known as vitamin B_9_, is one of the most largely consumed nutrient supplements by the general public. Folate provides the one-carbon units for the biosynthesis of many macromolecules, including amino acids, proteins, nucleotides, neurotransmitters and *S*-adenosylmethionine (SAM). SAM is the major methyl-donors for most methylation reactions of intracellular molecules, including DNA, RNA, histones, proteins and lipids, justifying the pivotal roles of folate in modulating gene activities and molecular functions (Boeke et al., 2012; Barua et al., 2014; Tryndyak et al., 2016; Morscher et al., 2018). Reduced folates are also strong anti-oxidants crucial to maintaining intracellular oxidative stress and embryogenesis (Rezk et al., 2003; Gliszczynska-Swiglo, 2007; Kao et al., 2014). The multi-activities and crucial roles of folate allow this nutrient to become a vital element for supporting normal physiological function and a favorite target for chemotherapy regimen. FD is one of the most commonly encountered problems in malnutrition. FD has also been linked to many diseases and anomalies, including neural tube defects (NTD), cancers, cardiovascular diseases, liver diseases, age-related cognitive impairments, and eyes malformation (Ducker and Rabinowitz, 2017). The growing awareness of the importance of folate has increased the public demand for folate supplementation although the underlying mechanism for folate-related diseases remains incompletely understood. The ease of public access to folate also endows this nutrient a modifiable environmental factor and a nutraceutical that can modulate both health development and disease onset. Ample amounts of folic acid (FA) are ingested by pregnant mothers, the senior population and the general public as a daily supplement. The beneficial effects of folate supplementation and food fortification have been well documented (Gisondi et al., 2007; Field and Stover, 2018). However, the detrimental effects, such as interfered immunity, increased risk for tumorigenesis, infant bronchiolitis and childhood asthma, have also been reported (Whitrow et al., 2009; Veeranki et al., 2014). The conflicting results reported for folate supplementation have raised the concern and vigorous debate on the policy of mandatory folate fortification of food and maternal FA supplementation, a highly prevalent maneuver world-wide. Hence, investigations on how folate affects normal physiological functions and disease pathomechanisms become imperative and urgent (Dixit et al., 2018).

**FIGURE 1:**
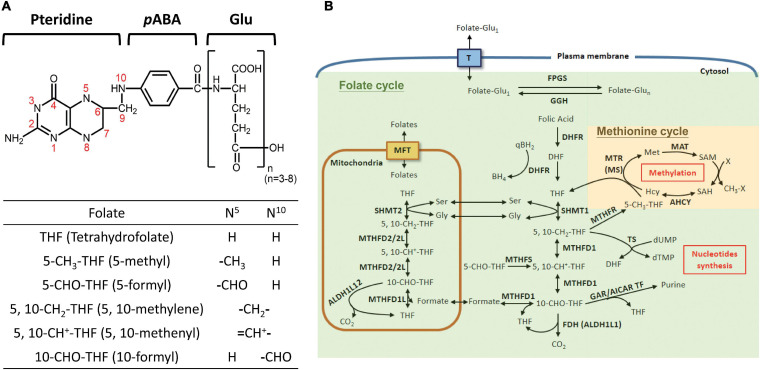
Folate-mediated one-carbon metabolism. **(A)** Structure of folate derivatives. **(B)** FOCM consists of multiple reactions and contributes to amino acid metabolism, nucleotide synthesis and SAM formation. The enzymes involved in FOCM include: FPGS, folylpolyglutamate synthase; GGH: γ-glutamylhydrolase; DHFR, dihydrofolate reductase, SHMT1, cytosolic serine hydroxymethyltransferase; SHMT2, mitochondrial SHMT; MTHFD1, cytosolic tri-functional enzyme, C1-THF-synthetase; MTHFD2/2L, mitochondrial bi-functional enzyme (5,10-methylene-THF dehydrogenase/5,10-methenyl-THF cyclohydrolase); MTHFD1L, mitochondrial 10-formy-THF synthetase; FDH, 10-formyltetrahydrofolate dehydrogenase; MTHFS, 5-formyltetrahydrofolate cyclo-ligase; MTR, 5-methyltetrahydrofolate-homocysteine methyltransferase; MAT, methionine adenosyltransferase; AHCY, adenosylhomocysteinase; MTHFR, 5,10-methylene-THF dehydrogenase; TS, thymidylate synthetase; GAR/AICAR TF, glycinamide ribonucleotide/aminoimidazole carboxamide ribonucleotide transformylase. T, folate transporters; MFT, mitochondrial folate transporter; the green area indicates the folate cycle, and yellow area indicates the methionine cycle.

Folate is comprised of a pteridine ring, *p*-aminobenzoic acid (*p*ABA) and glutamyl moieties (Figure 1A). FA refers to the synthetic and fully oxidized folate found in fortified food and supplements. The biologically active forms of folate are fully reduced tetrahydrofolate (THF), the activated intracellular one-carbon carriers and a strong antioxidant. In cells, folate exists as polyglutamate forms with 3–8 glutamate residues attached to the γ–carbon of the first glutamate residue. Nature folates are a mixture of THF carrying one-carbon units at the oxidation levels of formate, formaldehyde and methanol bound to THF at either the N-5 and/or N-10 positions, forming a large group of folate adducts. FOCM comprises two major folate-dependent biosynthetic networks, designated as folate cycle (for thymidylate and purine biosynthesis) and methionine cycle (for methionine/SAM formation) (Figure 1B), respectively. In general, each one-carbon adduct plays an indispensable role in one major pathway, i.e., 5-CH_3_-THF in SAM formation, 5,10-CH_2_-THF in dTMP synthesis, and 10-CHO-THF in purine production. Theoretically, disrupting any of these cycles results in auxotrophy for the end products and accumulation of the respective intermediates in that cycle. FOCM also occurs in mitochondria and nucleus. Mitochondrial FOCM converts one-carbon units to formate, which is exported and incorporated into 10-CHO-THF in cytosol or nucleus for purine biosynthesis (Sudiwala et al., 2016). Mitochondrial FOCM is the main source of one-carbon units yet the reversal of cytosolic flux can also be triggered under certain contexts to support cell growth (Ducker et al., 2016). Pharmacological inhibition of FOCM by targeting folate enzymes has been one of the mainstays for chemotherapy currently. However, the associated side-effects and often emerging drug-resistance have impeded the curative potential of anti-folates (MacKenna et al., 2020). Therefore, tremendous efforts have been devoted to investigating the biological activity of folate/folate enzymes, aiming to unveil the pathomechanisms underlying folate-associated disorders and develop new strategies/drugs with improved efficacy.

The diverse and conflicting effects among tissues reported for altered folate status imply the tissue heterogeneity of FOCM. FD enhanced invasiveness of colon cancer while blocking prostate cancer progression (Bistulfi et al., 2011; Wang et al., 2012). Folic acid supplementation prevented fetal NTDs but increased the risk of infant bronchiolitis and childhood asthma (Whitrow et al., 2009; Veeranki et al., 2014). In addition, FA supplementation increased the incidence of prostate cancer but was protective against head and neck squamous cell carcinoma, hepatocellular carcinoma and neuroblastoma (Strickland et al., 2013). Previous studies also showed that FA rescued only the cardiac malformation but not the impeded hematopoiesis in FD zebrafish (Tu et al., 2017). This tissue heterogeneity is a reflection of the intrinsic complexity of FOCM and suggests diverse pathomechanisms underlying the FD-induced pathology in different tissues. Investigations employing multiple platforms with complementary advantages for organ-specific studies shall provide new insights to understanding the physiological significance and pathological mechanism associated with FOCM.

In recent years, the use of zebrafish has gradually gained research interest from both academia and private companies to study various human diseases and patholomechanisms, such as host–pathogen interactions (Torraca and Mostowy, 2018), osteoporosis (Zang et al., 2021), nutritionology (Williams and Watts, 2019), immune response (Xie et al., 2020), and obesity-related diabetes (Zang et al., 2017, 2019), and to develop novel drug delivery systems for cancer treatments (Shimada et al., 2014; Shimada and Hazekawa, 2021). Zebrafish is a newly emerging vertebrate model and shares considerable structural and functional similarities with mammals in their ocular systems, including layer distribution, predominant cone-mediated vision and developmental process (Link and Collery, 2015; Richardson et al., 2017). As also seen in mammals, zebrafish eye development incorporates surface ectoderm, neuroectoderm and head mesenchyme, proceeded under stringent spatiotemporal regulations (Schmitt and Dowling, 1999). The highly conserved genetic programs and mechanisms involved in zebrafish ocular development also display extensive molecular complexity (Glass and Dahm, 2004). Zebrafish express photoreceptors that are spatially arranged in a highly organized mosaic architecture similar to the human counterpart (Richardson et al., 2017). In addition, their cone-rich eyes fulfill the diurnal nature of zebrafish and enable the fish to discriminate red, green, blue, and UV wavelengths (Allison et al., 2004). Zebrafish are visually responsive by 72 hour-post-fertilization (hpf) and display a variety of locomotor behaviors in response to moving patterns with brightness, sharpness, chromaticity and resolution, which can be varied depending on the components of the visual system under investigation (Fleisch and Neuhauss, 2006). Zebrafish possess a prominent binocular structure which is the most observable organ among all, especially at the larvae stages, allowing for continuous and non-invasive observation throughout ocular development. More importantly, several visual- and ocular disorders, including glaucoma, albinism, retinitis pigmentosa, coloboma, myopia and ciliopathies, have been established in zebrafish (McMahon et al., 2004; White et al., 2008; Veleri et al., 2012; Carnes et al., 2014; Collery et al., 2014; Link and Collery, 2015), supporting the suitableness of using zebrafish to model ocular disorders. Previous studies have shown that zebrafish FOCM is comparable to mammalian counterparts for the activities, functions and regulations of many enzymes/proteins involved in this metabolic network (Chang et al., 2006; Chang et al., 2007; Kao et al., 2008, 2009, 2014; Lin et al., 2011, 2015; Chang et al., 2014; Hsiao et al., 2014).

In the current study, we investigated the underlying patho-mechanism of FD-induced microphthalmia and the effectiveness of supplementing with different folate adducts using the fluorescent transgenic zebrafish, Tg(*hsp*:EGFP-γGH), as an *in vivo* model. This transgenic line displays inducible FD and ocular defects. The impact of FD to FOCM homeostasis and the differential regulation of folate enzymes were also inspected. In addition, the interplay between folate and vitamin A metabolism in eye development were also reported.

## Materials and Methods

### Material

Tetrahydrofolate and 5-methyl-THF were gifts from Dr. Moser (Merck Eprova AG, Switzerland). 5-formyltetrahydrofolate was purchased from Schircks Laboratories (Bauma, Switzerland). Fetal bovine serum (FBS) and trypsin-EDTA were purchased from Invitrogen, Thermo Fisher Scientific Inc. (CA, United States). dNTP was purchased from FocusBio (CA, United States). *In vitro* transcription kit, anti-DIG antibody, and NBT-BCIP used for WISH were purchased from Roche (Basel, Switzerland). Antibodies for immunostaining were purchased from either Santa Cruz Biotechnology Inc. (DA, United States) or Abcam plc. (Cambridge, United Kingdom). All other chemicals were purchased from Sigma-Aldrich Chemical Co. (WI, United States). The HPLC gel filtration column AQUASIL C18 was purchased from Thermo Fisher Scientific Inc. (MA, United States).

### Fish Stock and Maintenance

Wild-type (AB strain) zebrafish were purchased from Taiwan Zebrafish Core Facility (TZCS). Tg (*hsp*:EGFP-γGH), the transgenic line displaying inducible FD, was established in our lab (Kao et al., 2014). Fish were maintained in a 14-10 light-dark cycle at 25–28°C. All the operational and experimental procedures, including feeding, maintenance, mating, and sample collection, were approved by the Affidavit of Approval of Animal Use protocol of National Cheng Kung University (IACUC Approval Number: 103218 and 106086).

### Induction of Folate Deficiency

The transgenic embryos/larvae with various severity of FD were generated and categorized as previously described (Kao et al., 2014). In brief, Tg(*hsp*:EGFP-γGH) embryos were heat-shocked at 9 and 24 hpf at 38–39°C for 1 h each time and categorized into control group (CTL, with no fluorescence), mild folate-deficient group (MFD) and severe folate-deficient group (SFD) based on their fluorescence intensity at 32 hpf and total folate content at 5 day-post-fertilization (dpf). We had shown previously that the extent of FD was positively correlated to heat-shock and green fluorescence intensity.

### Histochemical Staining and Eye Size Measurement

For eye size measurement, larvae were photographed laterally under a dissecting microscope (Leica) equipped with a digital camera (Canon, EOS 550D). The eye area in the image was circled individually and calculated with the on-line software Image J^1^. For lens length measurement and detailed morphology, the cryo-sections prepared from the embryos/larvae at indicated stages were H&E stained and observed under a light microscope. The length of lens was measured only from those tissue sections containing the optic nerve to ensure the measurement for the full-length diameter.

### Whole-Mount Immunostaining

The whole-mount immunostaining for proliferating cells was performed following the protocol described by others (Thisse et al., 1993; Jowett, 2001). In brief, embryos were fixed in 4% PFA/PBS and permeablized with acetone in –20°C. Embryos were incubated with mouse monoclonal anti-phosphohistone H3 antibodies (1:200) and goat anti-mouse IgG Alexa Flour^®^ 488 (1:400) sequentially with proper wash and observed under a fluorescent dissecting microscope. The number of PH3+ cells within eye area were quantified by counting the cells with fluorescent signal with the on-line software Image J.

### TUNEL Assay

Fluorescein-labeling for apoptotic cells in larvae was performed with *in situ* Cell Death Detection Kit, following the manufacturer’s instruction (11684795910 ROCHE, Sigma-Aldrich Inc.). After permeabilization with acetone in –20°C, larvae were incubated with the mixture of enzyme solution (containing TdT) and labeling solution (containing fluorescein-dUTP) for an hour before photographed under a fluorescent dissecting microscope. The number of apoptotic cells within eye area were quantified by counting the cells with green fluorescent signal using the on-line software Image J.

### Quantification for Gene Expression

For determining gene expression, RT-PCR, real-time PCR and Western blotting were performed as previously described (Chang et al., 2010). In brief, RT-PCR was performed following the procedure of Prime Taq^TM^ DNA Polymerase (G-1001-1, GENET Bio, Chungnam, South Korea) with Applied Biosystems^TM^ SimpliAmp^TM^ Thermal Cycler (ThermoFisher Scientific, MA, United States). Real-time PCR was performed following the manufacturer’s instructions of KAPA SYBR^®^ FAST qPCR Master Mix (2X) Kit (KK4600, KAPABIOSYSTEMS, Cape Town, South Africa) and LightCycler (Roche Diagnostics, Rotkreuz, Switzerland). The sequences of primers used in the current study were listed in Supplementary Table 1.

### Embryonic Folate Content

Larval folate contents were determined as previously described (Kao et al., 2013). In brief, approximately 50 embryos were homogenized in 0.3 ml of de-gassed extraction buffer [100 mM Kpi, 2% (w/v) Ascorbic acid, 0.1% (v/v) 2-Mercaptoehtanol, pH6.8] and heated at about 98°C for 5 min before centrifugation. Conversion of folyl polyglutamates in the supernatant to folate monoglutamates was accomplished by incubating the embryos extracts with 5 μg of purified recombinant zebrafish gamma-glutamyl hydrolase (GGH) and at 37°C for 5 min. After centrifugation and filtration, 50 μl of the clear supernatant was injected into an AQUASIL C_18_ column on an HPLC system (Agilent 1100) for folate detection. The potential folate peaks in extracts were identified by overlapping the retention times between the prospective folate peaks and folate standards.

### Cloning for Zebrafish *mthfd1L*

Full-length zebrafish mitochondrial methylenetetrahydrofolate dehydrogenase (*mthfd1L*) cDNA was PCR amplified from zebrafish cDNA library with primers (forward: 5′-CCCC G AATTC ATGAT GAGAC TGTCT GTC-3′/ reverse: 5′-TGCGG CCGCA CGAAA AGGCC TTTTA TCTC-3′) designed based on mRNA sequence available in the Nucleotide database in NCBI (NM_001242996.1). The resulting PCR product was sub-cloned into pcDNA3.1 myc-His vector via *Eco*RI and *Not*I restriction sites to fuse with His-tag. This zebrafish *mthfd1L* coding sequence was also sub-cloned to pcGlobin2 for capped mRNA synthesis with another pair of primers (forward: 5′-CCCCG AATTC ATGAT GAGAC TGTCT GTC-3′/ reverse: 5′-CGAAT TCTCA ATGGT GATGG TGATG ATG-3′).

### MTHFD1L Knockdown and Rescue

The knockdown and rescue for *mthfd1L* expression were performed as previously described with modification (Chang et al., 2014). The pcGlobin2 plasmids containing complete zebrafish *mthfd1L* coding sequence were linearized with *Nhe*I and *in vitro* transcribed using T7 polymerase following the manufacturer’s protocol (10 881 767 001, Roche). The morpholino oligonucleotides (MOs) specific to zebrafish *mthfd1L* mRNA splicing site (5′-AAAGTAA AGCACACCTTTGACTCCA-3′) and translation initiation site (5′-AAGGCGACAGACAGTCTCATCATGC-3′) to knockdown the expression of zebrafish *mthfd1L* were designed and purchased from Gene-Tools (Philomath, OR, United States). All reagents for microinjection were dissolved in degassed and RNase-free Danieu’s buffer before injection. For microinjection, approximately 125–250 pg of MO with/without *mthfd1L* capped mRNA in 4.6 nl injection solution were injected into embryos at 1–4-cell stages. Embryos injected with Danieu’s buffer and standard control MO served as injection controls in each experiment.

### Primary Culture of Embryonic Cells

The protocol for preparing the primary culture from zebrafish embryos was adapted from previous studies with modification (Horstick et al., 2013). Briefly, the control and the embryos injected with capped mRNA encoding His-tagged MTHFD1L at 1–4-cell stage were collected at 1 dpf and dechorionated. After dissociating with trypsin-EDTA, embryonic cells were washed and filtered through a 70 μm strainer in L15 medium before seeded on the cover slides coated with poly-L-lysine in a 24-well plate. Cells were incubated at 28.5°C without CO_2_ for 12–16 h before immunofluorescent staining.

### Characterization for the Cellular Compartmentation of Recombinant Zebrafish MTHFD1L

The mitochondrial compartmentation of recombinant zebrafish MTHFD1L-His fusion protein was characterized by immunostaining with anti-His antibodies and co-staining with Mitotracker Deep Red 633 (Invitrogen) and DAPI for mitochondria and nuclear localization, respectively, as previously described (Chang et al., 2007).

### Compound Treatment

Compounds, including 5-formyl-THF (0.5 mM), FA (1 mM), 5-methyl-THF (1 mM), retinoic acid (1 nM), *N*-acetyl-L-cysteine (20 μM), sodium formate (5 mM), and dNTP (50 μM), were freshly prepared in embryo water, added to embryo water 30 min after heat-shock and refreshed every day until observation.

### Optomotor Response (OMR)

Evaluation for larval OMR was performed following the protocol adapted from previous studies with modification (Antinucci and Hindges, 2016). In brief, the individual larva was initially positioned at the center of a 6-cm petri dish placed on an iPad displaying moving black-white vertical gratings. Normally, larva swims along the direction of moving gratings and turns when the marching direction of moving gratings was changed. The swimming behavior of each larva was recorded while the direction of moving strips was reversed between right- and left-ward every 60 s for 2 min. Larvae that failed to respond to the reversal of strips movement were considered abnormal.

### Mitochondria Fractionation and Western Blotting

Mitochondria fractionation of cultured human hepatocellular carcinoma Huh-7 cell-line was performed following the protocol described previously with modification (Villa-Cuesta and Rand, 2015). Cells transfected with plasmids zmthfd1L-His-tag/pcDNA3.1 were collected, washed with buffer A [100 mM sucrose, 1 mM EGTA, 20 mM MOPS] and lysed in buffer B [100 mM sucrose, 1 mM EGTA, 20 mM MOPS, 5% Percoll, 0.01% digitonin] containing protease inhibitors. Cell lysates were centrifuged at 800 *g* for 10 min at 4°C. The supernatant was collected and centrifuged again at 20,000 *g* for 20 min. The resulting supernatant was collected separately for the cytosolic fraction. The pellet was suspended in buffer C [300 mM sucrose, 1 mM EGTA, 20 mM MOPS, 1% TritonX-100] for the mitochondrial fraction. Both fractions were subjected to Western blotting probing with antibodies against His-tag, α-tubulin or Cox4.

### Statistical Analysis

The probability value (*P*-value) was calculated with Student’s *t*-test and Mann–Whitney non-parametric *U* test for the rest at 95% confidence intervals using software GraphPad Prism 5.

## Results

### FD Caused Microphthalmia and Impeded Visual Ability in Developing Larvae

**FIGURE 2:**
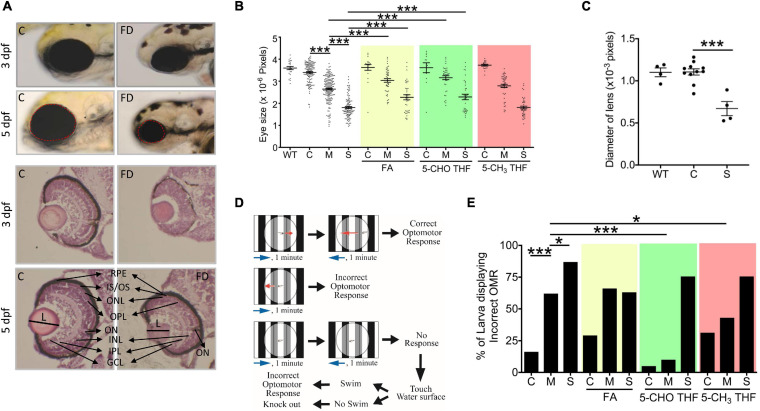
Morphological and functional characterization on the eyes of FD larvae. **(A,B)** Control and FD larvae at indicated stages with/without folate supplementation were imaged laterally for the head region and quantified for the eye area (dotted circle) with Image J (An on-line software for scientific image analysis.). The larvae in folate supplemented groups were exposed to the indicated folate adducts at 25 hpf and until observation. **(C)** The diameter of lens was estimated from the images of H&E stained cryo-sections prepared from the larvae at 5 dpf. **(D)** The experimental flow of optomotor response analysis (OMR). **(E)** Control and FD larvae with/without folate supplementation were analyzed for OMR at 7 dpf individually and quantified. WT, wild-type; C (control), heat-shocked wild-type larvae; M, FD transgenic larvae with mild FD. S, FD transgenic larvae with severe FD. L, length of lens; ON, optic nerve; RPE, retinal pigmented epithelium; IS/OS, inner segment/outer segment junction; ONL, outer nuclear layer; OPL, outer plexiform layer; INL, inner nuclear layer; IPL, inner plexiform layer; GCL, ganglion cell layer; FA, folic acid (1mM); 5-CHO THF, 5-formyltetrahydrofolate (0.5 mM); 5-CH_3_ THF, 5-methyltetrahydrofolate (1 mM). Statistical data are shown in mean ± SEM. ***p-value < 0.001; **p-value < 0.01; *p-value < 0.05.

We found that FD larvae displayed prominent smaller eyes. Larval eye size was measured by quantifying the dimension of eye area in the larval images photographed at 3 and 5 dpf (Figure 2A). A significant decrease in the overall eye size and lens diameter was observed in FD larvae beyond 3-dpf in a FD severity-dependent manner, as compared with the control larvae of the same stages (Figures 2A–C). This reduced eye size was prevented by FA and 5-CHO-THF supplementation, supporting the causal specificity of FD (Figure 2B). Intriguingly, no apparent improvement occurred when 5-CH_3_-THF was used for rescue. Histological characterization on the H&E stained tissue cryo-sections revealed unlaminated eyes in FD larvae at 3 dpf, in contrast to the apparent lamination observed in control larvae (Figure 2A). Larval visual function was accessed at 7 dpf by monitoring their optomotor response (OMR). OMR is an organismal and innate reflex to moving gratings stimulation commonly seen in the animal kingdom (Figure 2D). In zebrafish, OMR has been used to evaluate visual acuity, which reflects the function of the visual system (Orger et al., 2004). An approximately 5–6-fold increase in the occurrence of incorrect OMR was observed in the FD group in a FD severity-dependent manner (Figure 2E). Supplementing with either 5-CHO-THF or 5-CH_3_-THF, but not FA, significantly improved the visual response of FD larvae, confirming the causal role of FD. These results showed that FD induced ocular malformation and visual defects in developing embryos, as well as the diverse rescuing effects of different folate adducts.

### FD Affected Cell Proliferation and Induced Apoptosis in a Developmental Timing-Specific Manner During Embryogenesis

**FIGURE 3:**
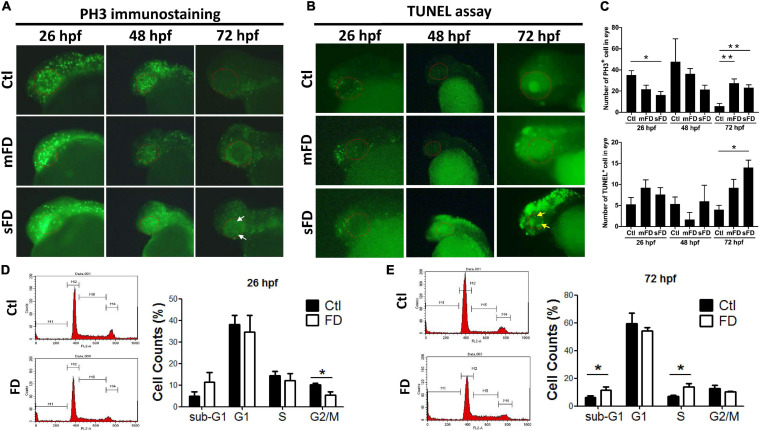
Developmental timing-specific analysis for the cell proliferation and cell cycle in developing larvae. The larvae of control and FD groups at 26, 48, and 72 hpf were subjected to immunostaining with anti-PH3 antibodies (M-phase marker) **(A)**, as well as TUNEL assay for apoptosis **(B)**. The number of PH3+ cells and apoptotic cells within eye area were quantified by counting the cells with fluorescent signal using the on-line software Image J **(C)**. Larvae at 26 hpf **(D)** and 72 hpf **(E)** were dispersed, stained with propidium iodide (PI) and analyzed for cell cycle with flow-cytometry and quantified. White arrows indicate the signals of PH3-positive cells; yellow arrows represent the signals of apoptosis; red dotted lines circle the eye area. Statistical data are shown in mean ± SEM. **p-value < 0.01; *p-value < 0.05.

Decreased cell proliferation at the very early stages and increased apoptosis at later stages of embryonic development were observed in FD larvae. The decreased eye size prompted us to examine embryonic cell proliferation with whole-mount immunostaining for phospho-histone 3 (PH3), a mitosis-specific marker. The results showed that there were fewer positive signals (represented by green fluorescent dots, which shall be distinguished from the green fluorescent background resulting from the expressed recombinant eGFP-γ-glutamyl hydrolase) in FD larvae than those in control before 48 hpf, especially in the head region and eyes. The PH3 signals in FD larvae became apparent at 72 hpf (Figures 3A,C). The results of TUNEL assay showed no significant difference between control and FD embryos before 48 hpf, yet the apoptotic signals increased in the forebrain and retina of FD larvae at 72 hpf (Figures 3B,C). Cell cycle analyses revealed decreased distribution of cells in G2/M phase at 26 hpf and accumulation in S-phase and sub-G1 phase at 72 hpf in FD larvae (Figures 3D,E). These results suggested that FD interrupted cell cycle at very early embryonic stages and increased apoptosis, especially at larval anterior, as embryogenesis proceeded.

### FD Interfered With One-Carbon Metabolic Homeostasis and Nucleotides Formation

**FIGURE 4:**
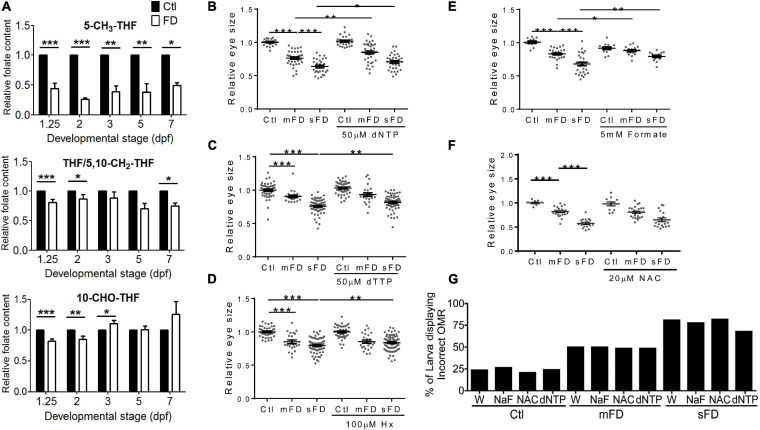
Larval folate composition and response to rescuing agents. Both control and FD embryos continuously exposed to rescuing agents starting from 25 hpf were examined for their intracellular folate composition **(A)** at indicated stages with HPLC as described in materials and methods, eye size **(B–F)** at 3 dpf and OMR **(G)** at 7 dpf. The rescuing agents include deoxy-ribonucleoside triphosphate (dNTP) **(B)**, Deoxythymidine triphosphate (dTTP) **(C)**, hypoxanthine (Hx) **(D)**, formate (NaF, sodium formate) **(E)** and *N*-acetyl-cysteine (NAC) **(F)**. Statistical results are shown in mean ± SEM for **(B–F)** and percentage in a group for **(G)**. ***p < 0.001; **p < 0.01; *p < 0.05.

The incoherent and developmental timing-specific fluctuation of different folate adducts were observed in FD larvae. Embryos of various stages were analyzed with HPLC for the composition of folates, mainly THF/5,10-CH_2_-THF, 10-CHO-THF, and 5-CH_3_-THF. THF is the main one-carbon carrier moiety; 5-CH_3_-THF participates in *S*-adenosylmethionine formation and hence crucial to modulating intracellular methylation potential; 10-CHO-THF provides the formyl group for cytosolic one-carbon source and the C2 and C8 of pteridine ring in purine biosynthesis (Bryant et al., 2018). Our results showed that the levels of all the examined folates were decreased after the second heat shock at 24 hpf to induce FD (Figure 4A). As embryogenesis proceeded, the concentrations of 5-CH_3_-THF continued to stay significantly low throughout the embryogenesis. Conversely, THF/5,10-CH_2_-THF content in FD larvae was increased gradually but remained lower than that of control larvae. As for 10-CHO-THF, the initial decrease was followed by a small but significant increase when the larvae reached 3 dpf and then maintained at a level comparable to that of control. Adding dNTP, dTTP and hypoxanthine to embryo water significantly improved larval eye size (Figures 4B–D). Supplementing with formate also rescued larval eyes size (Figure 4E). Formate is a major source for one-carbon unit in cytosol (Momb et al., 2013; Brosnan and Brosnan, 2016). These results suggested that FD disturbed OCM homeostasis and impeded nucleotide formation. FD has been shown to increase intracellular oxidative stress in both cultured cells and developing embryos (Kao et al., 2014). *N*-acetylcysteine (NAC) is a commonly used anti-oxidant in the laboratory to prevent increased oxidative stress. We observed no rescue effect on the eye size when NAC was added to the developing FD embryos (Figure 4F). In addition, neither of the aforementioned supplementations provided protective effects for larval OMR (Figure 4G). Together with the decreased numbers of mitotic cells at 26 hpf and increased cell accumulation in S-phase at 72 hpf (Figure 3), these results suggested that FD disturbed OCM homeostasis and impeded nucleotide supply, contributing to FD-induced microphthalmia. In addition, other factors related to FD, besides the insufficient nucleotides supply, might also contribute to the FD-induced impairment of eye development and OMR. Our results also show that the intracellular content of 5-CH_3_-THF is the most responsive folate species among the examined folate adducts and significantly decreased when FD occurred.

### Mthfd1L Expression Was Increased in FD Larvae

**FIGURE 5:**
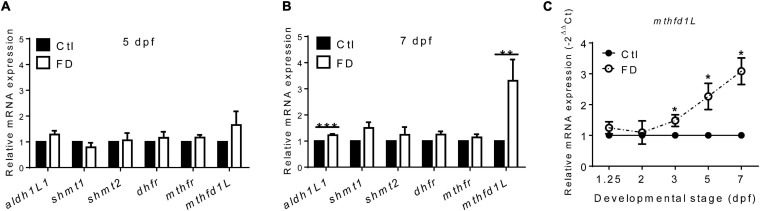
The mRNA levels of enzymes involved in FOCM in larvae. **(A)** FD larvae harvested at 5-dpf were subjected to mRNA extraction and RT-PCR analysis for the expression of folate enzymes. **(B)** Larvae displaying FD induced by heat-shock at 1, 3, and 5 dpf were harvested at 7-dpf and examined for the expression of folate enzymes with RT-PCR. **(C)** FD larvae collected at the indicated stages were examined for the developmental timing-specific expression of *mthfd1L* during embryogenesis with real-time PCR. Ctl, control; FD, folate deficiency; *aldh1l1*, zebrafish 10-formyltetrahydrofolate dehydrogenase; *shmt1*, cytosolic serine hydroxymethyltransferase; *shmt2*, mitochondrial serine hydroxymethyltransferase; *dhfr*, dihydrofolate reductase; *mthfr*, methylenetetrahydrofolate reductase. ***p-value < 0.001; **p-value < 0.01; *p-value < 0.05.

The incoherent fluctuation of folate adducts in response to FD in embryos at various stages suggested diverse regulation for the production/consumption of different folate adducts, prompting us to examine the expression of folate enzymes involved in OCM. The enzymes examined include: 10-formyltetrahydrofolate dehydrogenase (*aldh1L1*), cytosolic and mitochondrial serine hydroxymethyltransferase (*shmt1* and *shmt2*), dihydrofolate reductase (*dhfr*), 5,10-methylenetetrahydrofolate dehydrogenase (*mthfr*) and methylenetetrahydrofolate dehydrogenase (NADP^+^-dependent)-1-like (*mthfd1L*). These enzymes are involved in either the production or usage of THF, 10-CHO-THF or 5-CH_3_-THF in cytosol or mitochondria (Figure 1A). With the exception of *mthfd1L*, no apparent difference in their mRNA levels was observed for all the enzymes examined between the larvae of FD and control groups at 5 dpf (Figure 5A). Substantial increases were observed for both *mthfd1L* mRNA and *aldh1L1* mRNA in the larvae with increased FD severity at 7 dpf (Figure 5B). Further confirmation for the increased expression of *mthfd1L* in FD larvae revealed a stage-dependent elevation with significant increases starting from 3 dpf (Figure 5C). MTHFD1L is a mitochondrial folate enzyme catalyzing the conversion of 10-CHO-THF into formate and crucial to the maintenance of cytosolic one-carbon units for purine biosynthesis and energy metabolism (Bryant et al., 2018). These results showed that *mthfd1L* was up-regulated in response to decreased intracellular folates.

### Overexpressing *mthfd1L* at Early Stages Increased Intracellular 10-CHO-THF Content and Improved FD-Induced Microphthalmia

**FIGURE 6:**
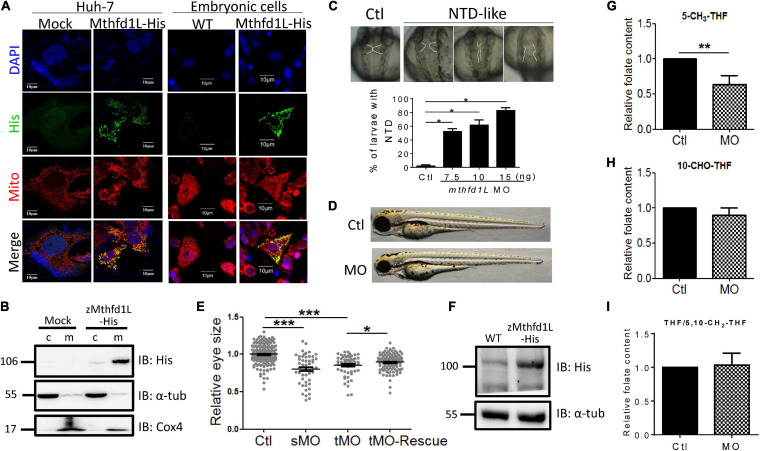
*In vitro* and *in vivo* characteristics of zebrafish *mthfd1L*. **(A)** The complete recombinant zebrafish Mthfd1L, encompassing the N-terminal signal peptide, was co-localized with mitochondria. Huh-7 cells transfected with z*mthfd1L*/pcDNA 3.1 myc-His (zMthfd1L) and pcDNA 3.1 myc-His (Mock) (left panel) and primary cultured zebrafish embryonic cells from embryos injected with *mthfd1L* cRNA (right panel) were stained with DAPI for nucleus, Mitotracker Deep Red 633 for mitochondria, and anti-His for zebrafish Mthfd1L. **(B)** The cytosolic and mitochondrial fractions of 293 cells transfected with z*mthfd1L*/pcDNA 3.1 myc-His and Mock analyzed with Western blotting confirmed the mitochondrial localization of zebrafish Mthfd1L. **(C)** The neural tube defect (NTD)-like phenotype was found in *mthfd1L* morphant (MO), but not in wild-type larvae (Ctl) at 3 dpf. **(D,E)** The eye size of *mthfd1L* morphants with (MO+cRNA)/without (MO) co-injecting synthesized *mthfd1L* capped-mRNA (cRNA) for rescue were examined and quantified at 3 dpf. Embryos were injected with translational MO (tMO) or splicing site MO (sMO) at 1–4-cell stage. **(F)** 1 dpf zebrafish embryos injected with *mthfd1L* cRNA were collected for embryonic lysates and subjected to Western blotting analysis. **(G–I)** *Mthfd1L* morphants at 3 dpf were analyzed with HPLC for folate composition. c, cytosolic fraction; m, mitochondrial fraction; α-tub, cytosolic marker; and Cox4, mitochondrial marker. MO, wild-type embryos injected with 10 ng mthfd1L MO; MO+cRNA, wild-type embryos co-injected with 10 ng mthfd1L MO and 1 ng mthfd1L synthesized RNA simultaneously. ***p < 0.001; **p < 0.01; *p < 0.05.

To examine the significance of Mthfd1L in embryonic morphogenesis, especially ocular formation, the complete cDNA encoding zebrafish Mthfd1L, including the N-terminal mitochondria-specific signal peptide, was cloned into eukaryotic expression vectors and expressed as a fusion protein with a C-terminal His-tag. This construct was used for characterizing the intracellular localization of zebrafish Mthfd1L in cultured cells and subsequent rescue in *mthfd1L* knockdown embryos. The results of immuno-staining revealed the co-localization of recombinant zebrafish Mthfd1L protein and Mitotracker, a mitochondria-specific dye, in Huh7 cells (Figure 6A). The co-localization of zebrafish Mthfd1L and Mitotracker was also observed in the primary cultured cells prepared from the zebrafish embryos injected with synthesized zebrafish *mthfd1L* mRNA. The subcellular compartmentation examined by cellular fractionation and Western blotting with anti-His-tag antibodies also supported the mitochondrial compartmentalization of the recombinant zebrafish Mthfd1L (Figure 6B). Morpholino oligonucleotides (MO) are anti-sense oligonucleotides often used to specifically knock-down the expression of interested genes in cells or organisms in the laboratory. *Mthfd1L*-specific MO interrupting either mRNA splicing or peptide translation were injected into wild-type embryos (morphants) at 1–4-cell stages. Malformed midbrain-hindbrain boundary appeared in *mthfd1L* morphants at 28 hpf with phenotypic severity positively correlated to the injected MO doses (Figure 6C). Microphthalmia was also observed in morphants at 3-dpf stage (Figures 6D,E). The lack of proper antibodies against endogenous zebrafish Mthfd1L prevented us from examining the knockdown efficiency with conventional immuno-blotting. However, the presence of aberrant splicing products in the cDNA prepared from the morphants confirmed the successful knockdown (Supplementary Figure 1). The appreciable rescue with *mthfd1L* mRNA further supported the knockdown specificity (Figures 6E,F). Unexpectedly, analysis on the folate content from embryos at 3-dpf revealed apparently lowered 5-CH_3_-THF levels but no appreciable change in both 10-CHO-THF and THF/5,10-CH_2_-THF levels in *mthfd1L* morphants, as compared to control larvae (Figures 6G–I). These results confirmed the mitochondrial localization of zebrafish Mthfd1L and supported a pivotal role of this enzyme in neural tissue development and ocular formation. Our data also suggested that 5-CH_3_-THF was the folate adduct most profoundly affected by the disturbed OCM due to *mthfd1L* knockdown.

**FIGURE 7:**
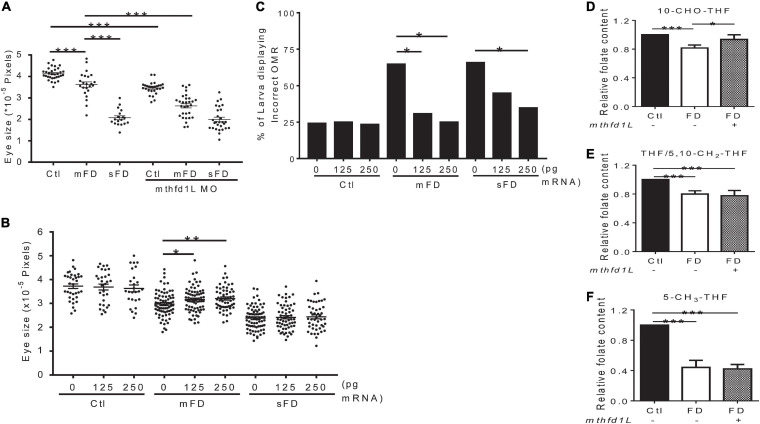
Effects of altering *mthfd1L* expression on eye development in folate-deficient embryos. **(A)** FD *mthfd1L* morphants had their eye size measured at 3 dpf. **(B)** FD embryos were injected with 0–250 pg zebrafish *mthfd1L* cRNA and their eye size was measured at 3 dpf. **(C)** *mthfd1L*-cRNA-injected control and FD embryos had their OMR estimated at 7 dpf. **(D–F)** 30 hpf control and FD embryos with or without 250 pg *mthfd1L* cRNA injection were subjected to HPLC analysis for their content of 10-CHO-THF **(D)**, 5-CH_3_-THF **(E)**, and THF/5,10-CH_2_-THF **(F)**. Ctl, control; mFD, mild folate deficiency; sFD, severe folate deficiency; *p < 0.05; **p < 0.01; ***p < 0.001.

Our results showed that increased *mthfd1L* expression in FD larvae prevented ocular defects. To examine the impact of altering *mthfd1L* expression in the occurrence of FD-induced ocular defects, FD embryos injected with either *mthfd1L* MO or mRNA at 1–4 cell stages were examined for ocular development. The results showed that knocking-down *mthfd1L* worsened eye malformation (Figure 7A); whereas increasing *mthfd1L* expression at early stages before FD induction prevented microphthalmia in the mild-FD group (Figure 7B). Substantial rescue for OMR was also observed in FD larvae injected with *mthfd1L* mRNA (Figure 7C). Unexpectedly, increasing *mthfd1L* expression prevented the decrease of 10-CHO-THF content in FD embryos but rendered no detectable impact on the restoration of the THF/5,10-CH_2_-THF and 5-CH_3_-THF levels (Figures 7D–F). These results supported the contribution of *mthfd1L* in maintaining the intracellular 10-CHO-THF level and eye development.

### The Expression of Methylenetetrahydrofolate Reductase (*mthfr*) Responses to FD at Early Stages

**FIGURE 8:**
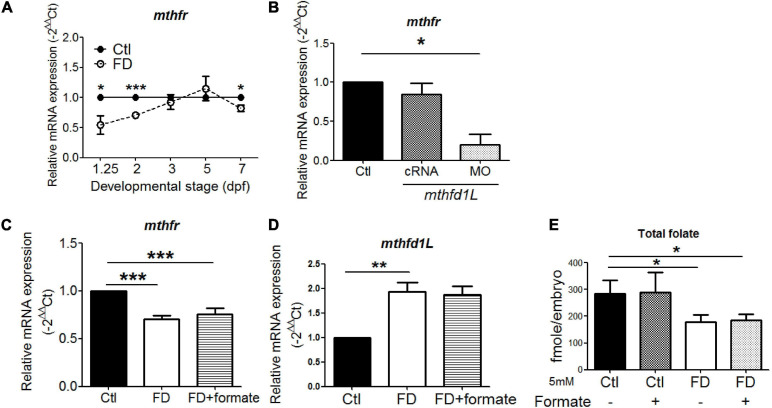
The interplay between Mthfr and Mthfd1L. **(A)** FD larvae collected at the indicated stages were examined for the developmental timing-specific expression of *mthfr* during embryogenesis. **(B)** Wild-type embryos injected with *mthfd1L* cRNA or MO were examined for the expression of *mthfr* at 1 dpf. **(C)** FD embryos with/without formate supplementation were examined for *mthfr* mRNA levels at 2 dpf. **(D)** FD embryos with/without formate supplementation were examined for *mthfd1L* mRNA levels at 5 dpf. **(E)** Control and FD embryos supplemented with or without 5 mM formate were measured for their total folate content at 2 dpf. ***p-value < 0.001; **p-value < 0.01; *p-value < 0.05.

The significant decrease of 5-CH_3_-THF level in both FD larvae and *mthfd1*L morphants prompted us to examine the expression of *mthfr*, the only folate enzyme catalyzing the irreversible production of 5-CH_3_-THF. Lowered expression of *mthfr* was found in FD embryos and *mthfd1L* morphants at earlier stages (Figures 8A,B). Providing one-carbon units by supplementing FD embryos with formate did not prevent the decrease of *mthfr* and the increase of *mthfd1L* in FD larvae (Figures 8C,D). The folate microbiological assay revealed an unaffected total folate content in embryos supplemented with formate (Figure 8E). This is not unexpected since FD was induced in our model by enhanced exportation of folate; whereas formate provides only one-carbon units but not folate moieties. Therefore, the situation of lowered total folate contents shall remain even in formate-supplemented FD embryos, so as the decreased and increased expression of *mthfr* and *mthfd1L*, respectively. Together, these results suggested that, in addition to the enhanced folate exportation upon FD induction, the decreased *mthfr* expression might further contribute to the constantly lowered 5-CH_3_-THF level observed in FD larvae.

### FD Interfered With Larval Visual Development

**FIGURE 9:**
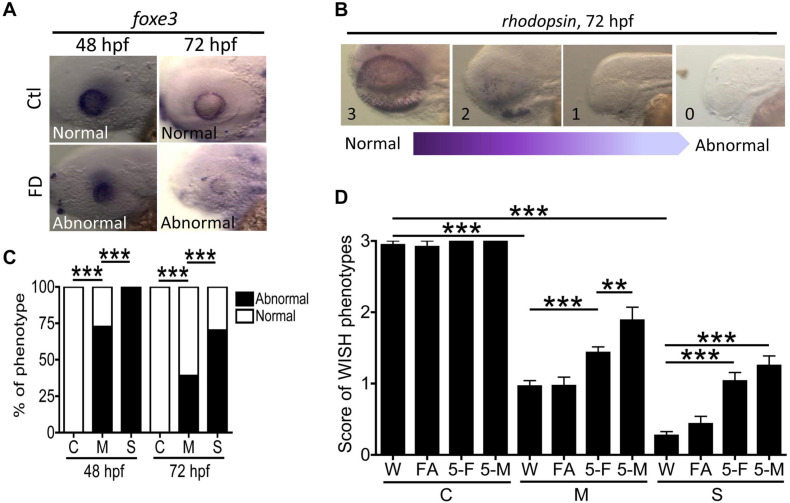
Analysis of ocular cell differentiation in FD larvae and response to folate derivatives. **(A,C)** Control and FD larvae at 48 and 72 hpf were characterized with WISH for the spatial distribution of *foxe3* (ocular lens marker) and quantified for the percentage of larvae displaying abnormal expression patterns. **(B,D)** The aberrant distribution of rhodopsin observed in the eyes of FD larvae revealed by WISH at 3 dpf were categorized into four scoring categories, based on the severity of anomalies displayed, with “3” to be normal and “1” to be complete lack of signal. The severity of abnormality was quantified by the averaged score of an anomaly for each larva observed for each experimental group, including those with/without folate supplementation. Data were obtained from at least three independent trials with the total sample numbers ranging from 7 to 80 for each group. C, control; M, mild folate deficiency; S, severe folate deficiency; FA, folic acid; 5-F, 5-CHO-THF; 5-M, 5-CH_3_-THF. Statistical data are shown in percentage of a group for **(C)** and mean ± SEM for **(D)**. ***p < 0.001; **p < 0.01.

The impaired retina lamination and OMR found in FD larvae suggested visual defects and prompted us to examine the cellular differentiation in eyes. WISH with the riboprobes specific to lens epithelium (*foxe3*) and rod cells (rhodopsin) was conducted with larvae at 48- and 72-hpf. In comparison to wild-type, FD larvae displayed weaker signal and irregular distribution patterns for lens epithelium in a FD severity-dependent manner (Figures 9A,C). Diminished signal for rhodopsin was also observed in FD larvae, which was significantly rescued by supplementing with 5-CHO-THF and 5-CH_3_-THF, but not FA, supporting the causal effects of FD (Figures 9B,D). We also noticed that 5-CH_3_-THF provided a better rescuing effect for the distribution of rhodopsin signals. These results suggested that FD impeded the development of lens epithelium and photoreceptor, contributing to the visual dysfunction in FD larvae.

**FIGURE 10:**
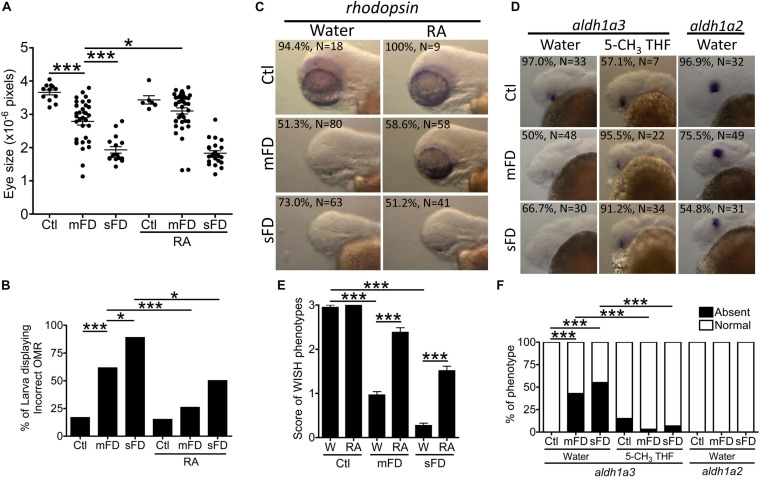
Ocular development in FD larvae and response to retinoic acid. Eye size **(A)** and OMR **(B)** of FD larvae with/without RA treatment were estimated at 5 and 7 dpf, respectively. The distributions of rhodopsin transcripts in larvae with/without RA supplementation were characterized with WISH at 3 dpf **(C)** and quantified **(E)**. The distribution of *aldh1a2* and *aldh1a3* transcripts in larvae with/without RA supplementation were characterized with WISH at 30 hpf **(D)** and quantified **(F)**. C, control; M, mFD; S, sFD. Statistical data are shown in mean ± SEM for **(A,E)** and percentage in a group for **(B,F)**. ***p < 0.001; *p < 0.05.

Our results showed that FD disturbed the expression of aldehyde dehydrogenase 1a3 (*Aldh1a3*), the enzyme catalyzing the formation of active vitamin A. Supplementing with retinoic acid improved eye size and OMR of FD larvae considerably (Figures 10A,B). Supplementing with retinoic acid also corrected rhodopsin expression found in FD larvae (Figures 10C,E). Retinoic acid is a vitamin A metabolite and a principal hormone required for a number of physiological processes, especially eye development. The successful rescue with retinoic acid suggested decreased availability of retinoic acid in FD embryos. The formation of all-*trans*-retinoic acid, the most active retinoid via retinal oxidation, is catalyzed by aldehyde dehydrogenases (ALDH). Meanwhile, *Aldh1a3* is the most efficient subtype among all ALDHs. WISH results revealed an exclusive expression of *aldh1a3* in the retina ventral of the lens in wild-type embryos at 30 hpf. Contrarily, the signal for *aldh1a3* in FD larvae was significantly decreased, which was rescued by 5-CH_3_-THF supplementation (Figures 10D,F). No apparent difference was seen for *aldh1a2* WISH signal between control and FD larvae. Our results suggested that the impaired retinoic acid supply, likely due to decreased *aldh1a3* expression, contributes to the obstructed eye differentiation in FD larvae.

## Discussion

**FIGURE 11:**
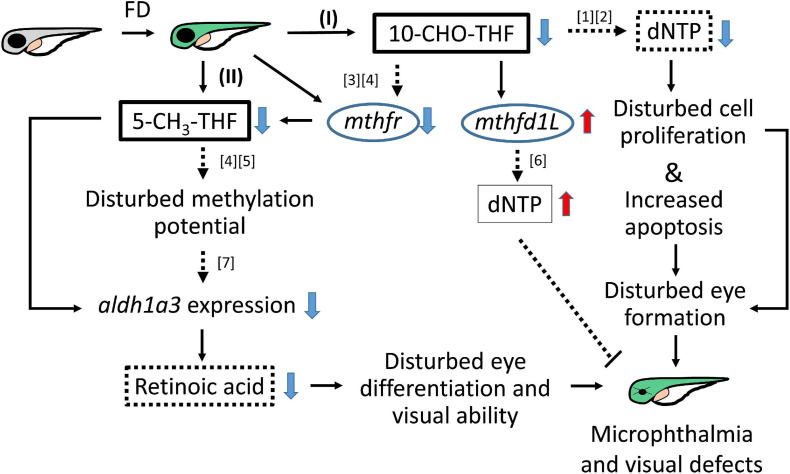
Proposed mechanism of FD-induced ocular defects. Solid lines indicate the experiments that were demonstrated in the current studies; dotted lines indicate the rationales supported by the listed references ([1] Henry et al., 2017; [2] James et al., 1994; [3] Leung et al., 2017; [4] Irwin et al., 2016; [5] Arasaradnam et al., 2008; [6] Bryant et al., 2018; [7] Duan et al., 2016).

Folate is crucial to a wide spectrum of biological processes, including gene activity control, cell proliferation and differentiation. Therefore, it is conceivable that multiple mechanisms are involved in FD-induced pathology, including maldevelopment of embryos. Our results revealed incoherent fluctuation among different folate adducts in response to disturbed FOCM in developing embryos, reflecting the contribution of multiple pathways to FD-induced ocular defects. Based on our results obtained in the current study and reported in the literature, a potential pathomechanism contributing to the FD-induced ocular defects was proposed, in which the in a developmental timing-specific manner - and context-dependent modulation of folate status and folate enzyme expression was involved (Figure 11). (I) The decreased intracellular folate obstructed dNTP supply initially, leading to the impeded cell proliferation at early embryonic stages, increased apoptosis at later stages and malformed eyes ultimately. Consequently, the expression of *mthfd1L* was upregulated, in response to the decreased folate and dNTP, in an attempt to replenish intracellular nucleotides pool and recuperate cell proliferation. (II) Intracellular 5-CH_3_-THF was decreased initially due to the increased recombinant γ-glutamylhydrolase activity and enhanced folate exportation. The expression of *mthfr* was also decreased, which further lowered the level of 5-CH_3_-THF in FD embryos, likely affecting the intracellular methylation potential and epigenetic control (Kao et al., 2014). As embryogenesis proceeds, *aldh1a3* expression was decreased, leading to impaired retinoic acid production, maldeveloped photoreceptors and visual defects. The phenotypic evidence and the potential pathomechanisms provided in the current study connect FOCM homeostasis to embryonic ocular development. The observations that different folate adducts offer differential rescuing effects for a specific FD-induced pathological phenotype imply context-dependent importance of different folate adducts. Our results also suggest that the causal mechanisms underlying the targeted diseases shall be taken into consideration while choosing a proper folate adduct for supplementation against a specific FD-related disease.

The differential rescuing effects provided by different folate adducts revealed irreplaceability among folate adducts. Folate adducts have usually been considered interconvertible among each other although they are different in their structures, properties and activities. FA is the most commonly consumed folate since it is included in folate fortified food and daily nutrient supplements. Adverse effects have been documented for long-term consumption of high-dose FA (Saitsu et al., 2003; Field and Stover, 2018). 5-CH_3_-THF is the most stable reduced folate. The advantage of supplementing with 5-CH_3_-THF had been reviewed, yet the enhanced toxicity of anti-folate compounds in the presence of 5-CH_3_-THF had also been reported (Kao et al., 2010; Scaglione and Panzavolta, 2014). 5-CHO-THF appeared to provide the best rescuing effects for both microphthalmia and visual dysfunction in FD larvae. 5-CHO-THF is also the folate supplement commonly prescribed for patients receiving anti-folate chemotherapy. Nonetheless, that decreased 5-CH_3_-THF level caused by methotrexate exposure in growing embryos could not be reversed by 5-CHO-THF supplementation also suggest the incompetence for a comprehensive rescuing effect with 5-CHO-THF (Kao et al., 2013). Therefore, proper caution shall be taken when folate supplementation and anti-folate drug are considered for preventive or therapeutic use. The diverse impact brought by folate supplementation also reflects the incompleteness of our knowledge on the biological properties and regulation of folate and FOCM. Further in-depth investigations on the impact of supplementing with different folate adducts are in need.

The incoherent fluctuation among different folate adducts in response to FD echoes the context-dependent and developmental timing-specific regulation of FOCM. Proper partitioning of one-carbon units among the reactions requiring one-carbon units are crucial to fulfill the metabolic needs under a specific physiological circumstance. The incoherent fluctuation among 5-CH_3_-THF, THF, and 10-CHO-THF, in terms of timing and composition, may provide embryos with the flexibility to deal with various stressful situations raised from different physiological needs (e.g., cell proliferation vs. differentiation) or pathological threats (e.g., compounds toxicity and malnutrition). It might also explain why inconsistent effects of folate supplementation were sometimes observed in clinical practice. For example, high seral folate levels were associated with a lower propensity for allergy, atopy, and wheezing; whereas maternal FA supplementation was reported for an increased risk of infant bronchiolitis and childhood asthma (Haberg et al., 2009; Whitrow et al., 2009; Barua et al., 2014). It was found subsequently that the timing and dose of maternal FA supplementation during pregnancy played a key role in modulating the occurrence of childhood asthma (Veeranki et al., 2015; Yang et al., 2015). Studies also showed that FA administered during an ethanol-sensitive time window provided better rescuing effects than administered after ethanol exposure for the ethanol-induced optic defects (Muralidharan et al., 2015). It is documented that mammals are capable of diverting endogenous formaldehyde into FOCM for detoxification and promoting nucleotides synthesis (Burgos-Barragan et al., 2017), also demonstrating the context-dependent regulation of FOCM in response to various physiological circumstances and distinct metabolic needs.

Our data suggest a role for Mthfd1L in nucleotides supply and ocular development. Proper cell proliferation is essential for organogenesis including ocular development. Impeding cell proliferation had been shown to interrupt optic fissure closure and ocular morphogenesis (Christensen et al., 2013; Momb et al., 2013). The initial decrease and then quick recovery of 10-CHO-THF levels in FD larvae occurred in consort with the increased expression of *mthfd1L*, implying a compensatory effect for the up-regulation of *mthfd1L*. This speculation is supported by the improvement and worsening of microphthalmia in embryos injected with the mRNA and MO to increase and decrease the expression of *mthfd1L*, respectively. It is also supported by the successful rescue with formate and dNTP for FD larvae. It is conceivable that Mthfd1L also affected methylation potential since knocking down *mthfrd1L* decreased 5-CH_3_-THF level, the folate required for *S*-adenosylmethionine formation. Our observations are consistent with the reports that Mthfd1L is crucial to both purine synthesis and methyl cycle (Bryant et al., 2018).

The intracellular 10-CHO-THF level is under stringent regulation and maintained within a stable and constant range. 10-CHO-THF is key to supporting nucleotide biosynthesis. Our results are in agreement with the reports that cells reversed cytosolic FOCM, in response to 10-CHO-THF depletion caused by *mthfd1L* knockout, to support cell growth and survival (Ducker et al., 2016). The up-regulation of *mthfd1L* could be a consequence of context-dependent gene regulation, in which the priority of nucleotides biosynthesis is maintained, likely in the expense of intracellular methylation potential.

MTHFR activity is crucial in controlling one-carbon flux in response to disturbed FOCM. We found that the intracellular 5-CH_3_-THF level is very sensitive to the depletion of intracellular one-carbon units. 5-CH_3_-THF is the most stable adduct among all reduced folates and crucial to SAM biosynthesis. Altered intracellular 5-CH_3_-THF content would profoundly affect cellular methylation potential and gene activity (Feng et al., 2017). Therefore, the high sensitivity of 5-CH_3_-THF level to intracellular folate content shall endow the cells with flexibility and capacity to deal with stress and stimulation by modulating genes activity quickly via methylation/demethylation to promote metabolic reprogramming and increase the cellular ability for adaption. 5-CH_3_-THF is generated in the unidirectional reaction catalyzed by MTHFR from 5,10-CH_2_-THF. Due to the irreversibility of reaction and “methyl-trap” associated MTHFR, this enzyme is crucial to modulate the partitioning of one-carbon units between methionine cycle (for methylation) and folate cycle (for nucleotide biosynthesis) (Leung et al., 2017). The decreased expression of *mthfr* observed in FD embryos throughout embryogenesis may act as a remedial measure to preserve one-carbon units within folate cycle for nucleotide formation, hence functionally coherent with the up-regulation of *mthfd1L*. Similarly, knocking-down *mthfd1L* in wild-type embryos, which led to decreased 5-CH_3_-THF, also decreased the expression of *mthfr*. Our results are in agreement with the studies showing that depletion of MTHFR helped retain the limited one-carbon units in folate cycle and echo the viewpoint that folate cycle for nucleotide biosynthesis was preserved at the expense of methionine cycle (Beaudin and Stover, 2009; Field et al., 2014; Leung et al., 2017). In addition, the interplay between *mthfd1L* and *mthfr* expression highlights the differential regulation of one-carbon units partitioning among cellular compartments and reactions. The metabolic reprogramming involving the differential regulation of *mthfr* and *mthfd1L* expression in response to FD exemplifies the context-dependent regulation of metabolic enzymes to prioritize the biochemical pathways vital for survival to support cellular and individual adaptability under stress.

The observations that RA significantly rescued FD-induced ocular defects evidences the link between folate and retinoic acid metabolism. We found that contrary to *aldh1a2*, which contains no potential methylation site, a CpG island has been identified in zebrafish *aldh1a3* promoter region (data not shown). This potential difference in the susceptibility to methylation regulation may provide the explanation for the different expression patterns between *aldh1a2* and *aldh1a3* in FD larvae, as well as the significant improvement in larval vision when 5-CH_3_-THF was used for rescue. The expression of human *ALDH1A3* was also reported to be associated with the methylation status of promoter in multiple malignancies (Shames et al., 2006; Kim et al., 2013; Marcato et al., 2015). In addition, several RA response elements (RARE) are identified in zebrafish *aldh1a3* promoter, suggesting an RA-mediated feedback loop, as reported for the expressional regulation of human *ALDH1A3* (Koenig et al., 2010). Our results supported the functional connection between folate and RA, as well as the evolutionary conservation of *ALDH1A3*.

## Data Availability Statement

The original contributions presented in the study are included in the article/Supplementary Material, further inquiries can be directed to the corresponding author/s.

## Ethics Statement

The animal study was reviewed and approved by The Affidavit of Approval of Animal Use protocol of National Cheng Kung University, Tainan, Taiwan (IACUC Approval Numbers: 103218 and 106086).

## Author Contributions

T-FF conceptualized this study and responsible for funding acquisition. T-HH, G-HL, and T-FF designed the experiments, analyzed the data, and wrote the manuscript. T-HH and G-HL performed the experiments. Y-SC and B-HC reviewed and provided consultancy for experimental design and data analysis. All authors contributed to the article and approved the submitted version.

## Conflict of Interest

The authors declare that the research was conducted in the absence of any commercial or financial relationships that could be construed as a potential conflict of interest.

## Footnotes

1 http://imageJ.net/ImageJ

## Supplementary Material

Supplementary Figure 1 Splicing MO efficiency test. **(A)** Scheme of potential PCR products of cDNA extracted from WT and *mthfd1L* splicing-interfering MO-injected embryos. **(B)** RT-PCR analysis of 1 dpf control and *mthfd1L* morphants, the major product was designated with white arrowhead for WT (product 1) and black arrow for *mthfd1L* morphants (product 2).

Supplementary Table 1 Primers used for RT-PCR and real-time PCR in this study.
