# Supplementary material for: The Incoherent Fluctuation of Folate Pools and Differential Regulation of Folate Enzymes Prioritize Nucleotide Supply in the Zebrafish Model Displaying Folate Deficiency-Induced Microphthalmia and Visual Defects
Source: Front Cell Dev Biol. 2021 Jun 29;9:702969. doi: 10.3389/fcell.2021.702969 (PMC8277299; doi:10.3389/fcell.2021.702969)
Supplement: Supplementary Table 1 — Primers used for RT-PCR and real-time PCR in this study. [file Data_Sheet_1.PDF]

Table S1. Primers used for RT-PCR and real-time PCR in the current study

Gene symbol	Accession No.	Primer sequence (5’- 3’)	Note
aldh1L1	NM_001198772.1	(F) CGCTGAGCATATGAGGGTGGTG<br>(R) GGTATAGACTGCTCCCGAG	
shmt1	NM_201046.1	(F) CCTATCAGTTGCAAG<br>(R) GAGCCAGTTCCTCC	
shmt2	NM_001123374.1	(F) GGAGAAGGTCAACTTC<br>(R) GCGATTCGAGAAACCG	
dhfr	NM_131775.1	(F) CAGAAGATGACCATGACCCCTTCAG<br>(R) GCTTGAGGATGCGGGTTACAAAC	
mthfr	NM_001281840.1	(F) ACAATGGGCGAAGCTATACG<br>(R) TAAGAAAACGCCACCCAAAC	
mthfd1L	NM_001242996.1	(F) GATATCGAGATCTCCAGAGCAC<br>(R) CCTCCATAGGAATGACCTGTG	
actb2	NM_181601.4	(F) AGACATCAAGGAGAAGCTGTG<br>(R) TCCAGACGGAGTATTTAC	
mthfr	NM_001281840.1	(F) GCTTTCGCGTTGTGGATT<br>(R) GTCCAGGAGCGCAAACAT	for real-time PCR
mthfd1L	NM_001242996.1	(F) GAAGGTTTACGGCGCTGA<br>(R) ACGTCTCTGATGGGCAGG	for real-time PCR
actb2	NM_181601.4	(F) GTCCACCGCAAATGCTTC<br>(R) ATTGCCGTCACCTTCACC	for real-time PCR
